# Enhanced Piezoelectric Properties and Conduction Mechanism in Na_0.5_Bi_2.5_Nb_2_O_9_ Piezoelectric Ceramics

**DOI:** 10.3390/nano15171293

**Published:** 2025-08-22

**Authors:** Jianming Deng, Kaijie Chen, Caijin Chen, Chenyang Zheng, Biao Zhang, Lanpeng Guo, Ting Wang, Kai Chen, Laijun Liu, Weiping Gong

**Affiliations:** 1 Guangdong Provincial Key Laboratory of Electronic Functional Materials and Devices, Huizhou University, Huizhou 516001, China; 2 Collaborative Innovation Center for Exploration of Nonferrous Metal Deposits and Efficient Utilization of Resources in Guangxi, Guilin University of Technology, Guilin 541004, China; 3 School of Integrated Circuits, Wuhan National Laboratory for Optoelectronics, Optics Valley Laboratory, Huazhong University of Science and Technology (HUST), Wuhan 430074, China; 4 School of Physics, Nanjing University of Science and Technology, Nanjing 210094, China

**Keywords:** Na_0.5_Bi_2.5_Nb_2_O_9_, conduction mechanism, oxygen vacancy, piezoelectric properties, thermal stability

## Abstract

In this work, (NaBi)_0.5−*x*_(LiSm)*_x_*Bi_2_Nb_2_O_9_ (NBN-*x*LS, *x* = 0.00–0.06) ceramics were fabricated by co-doping of LiSm into Na_0.5_Bi_2.5_Nb_2_O_9_. The traditional solid-phase technique was employed for the entire synthesis process. The impact of LiSm doping on the crystal structure, dielectric, ferroelectric, and piezoelectric properties, as well as the underlying conduction mechanisms in the NBN-*x*LS ceramics, was analyzed systematically. The XRD patterns and the Rietveld refinement revealed that lattice distortion reduced with an increase in the LiSm doping amount. The decrease in lattice distortion significantly contributed to its improved ferroelectric and piezoelectric characteristics. The results showed that the NBN-*x*LS ceramics were primarily *p*-type materials due to their bulk-limited conduction, with oxygen holes and vacancies acting as the conducting species, and the appearance of weak ion conduction at high temperatures. The NBN-0.04LS ceramic, in particular, displayed the highest performance, with *P*_r_, *T*_c_, and *d*_33_ values of 9.05 μC/cm^2^, 777 °C, and 25.2 pC/N, respectively. Additionally, the ceramic displayed remarkable thermal stability, with its *d*_33_ retaining 95.0% of its original value after annealing at 760 °C. These results demonstrate that LiSm co-doped Na_0.5_Bi_2.5_Nb_2_O_9_ ceramics have potential for use in high-temperature sensors.

## 1. Introduction

The rapid modernization of technology has led to a dramatic increase in demand for acceleration sensors that can operate at high temperatures in various industries, such as aerospace propulsion systems (75−500 °C), deep geothermal wells (450−650 °C), and other applications [1,2]. Therefore, the development of new and novel high-temperature piezoelectric materials is urgently required. Bismuth-layered ferroelectrics (BLSFs) are widely considered as potential contenders for use in the development of future high-temperature piezoelectric devices owing to their low aging rate, high Curie temperature, and excellent fatigue resistance [3]. BLSFs (also referred to as Aurivillius ferroelectrics) are ferroelectric compounds that were first reported by Aurivillius et al. The crystal structure of BLSFs consists of bismuth oxide (Bi_2_O_2_)^2+^ and perovskite-like layers (A_m−1_BmO_3m+1_)^2−^, alternately arranged in the *c*-axis direction [4]. The general formula for these materials can be defined as (Bi_2_O_2_)^2+^(A_m−1_B_m_O_3m+1_)^2^, given the arrangement of their crystal structures. The A-site, in general, is a large cation with a coordination number equal to 12, while the B-site is generally a small cation with a coordination number equaling 6. The integer *m* denotes the number of perovskite-like layers. The compound Na_0.5_Bi_2.5_Nb_2_O_9_ (NBN) is a typical member of the BLSF family, demonstrating a high Curie temperature (*T*_c_) of 780 °C with an *m* = 2 [5,6]. However, the piezoelectric constant (*d*_33_) for NBN is relatively low (~11 pC/N) due to its high structural anisotropy and large coercive field (*E*_c_). In addition, the defects induced due to the volatilization of Bi during the high-temperature sintering process impart an adverse effect on the high-temperature insulation [7]. The low resistivity at high temperature values can lead to charge drift that adversely interferes with the piezoelectric sensing charge, lowering the reliability and sensitivity of the piezoelectric sensors [2]. These limitations severely restrict its use in practical applications in the development of high-temperature electronic devices.

Considerable efforts have been made to mitigate this issue and improve the piezoelectric performance of NBN through various synthesis and optimization strategies, such as chemical modification and technological preparation modification, among others. For example, Aoyagi et al. reported the growth of single crystals of NBN from stoichiometric melts using a slow cooling method, which led to an increase in the electrical quality factor *Q*_m_ to 3800, far superior to its counterparts prepared through conventional approaches [8]. In addition, several studies have shown that the value of *d*_33_ in piezoelectric ceramics can be enhanced by improving the preparation technology, such as using spark plasma sintering [9]. However, the techniques required to improve the preparation processes are complex and challenging to implement. Therefore, the strategy of chemical modification, which is easier to execute and has also been shown to improve the performance of NBN, is being adopted extensively [10,11,12,13]. In a study by Zhou et al., the B-site Nb^5+^ ion of NBN was substituted by the W^6+^ ion, which nearly doubled the piezoelectric efficiency and enhanced the resistivity by two orders of magnitude [14]. In another study undertaken by Jie et al., it was reported that the dielectric loss was lowered along with an increase in the value of *d*_33_ to 20.1 pC/N upon substituting the B-site Nb^5+^ ion with a Co^3+^ ion [15]. In addition, the incorporation of Co/W at the B-site of the NBN ceramic led to an increase in the value of *d*_33_ from 10.5 to 24.9 pC/N, with good thermal stability [16]. Prior studies in the literature have reported that the piezoelectric characteristics of the NBN ceramics can also be improved by co-doping at the A-site. For example, it has been reported that the value of *d*_33_ increased to 25 pC/N when NaBi was partially substituted by LiLa at the A-site of the NBN ceramic. However, this led to a reduction in the value of *T*_c_ from 788 to 751 °C [5]. Another study found that the value of *d*_33_ in the Na_0.5_Bi_2.5_Nb_1.97_W_0.03_O_9_ ceramics enhanced from 21 to 26.1 pC/N upon the co-doping of LiCe at the A-site. However, the *d*_33_ value was found to be only 88.7% of its initial value after annealing at a temperature equal to 750 °C due to the high conduction currents present at high temperatures [17]. Unfortunately, there are few reports on Na_0.5_Bi_2.5_Nb_2_O_9_-based piezoelectric ceramics that can simultaneously satisfy the requirements of high *d*_33_ (>25 pC/N), high *T*_c_ (>770 °C), and excellent thermal stability.

Motivated by recent studies on the local structural heterogeneity and rare-earth doping of Pb(Mg_1/3_Nb_2/3_)O_3_-PbTiO_3_ [18] and Pb(Zn_1/3_Nb_2/3_)O_3_-PbTiO_3_ [19] ferroelectric single crystals, we introduce local structural heterogeneity through Sm^3+^ dopants to enhance the piezoelectricity of NBN ceramics. After the A-site is substituted with Sm^3+^ ions, the *T*_c_ usually decreases, accompanied by a change in the mean A-site ion radius. Li ions (0.76 Å, CN = 6; electronegativity: 1.0) have a higher electronegativity and smaller ionic radius than those of Na ions (1.39 Å, CN = 12; electronegativity: 0.9), which generally reduces the tolerance factor and symmetry of the compounds, potentially stabilizing *T*_c_. Therefore, it is hoped that the *d*_33_ value and thermal stability can be improved without reducing the *T*_c_ value through the co-doping of LiSm at the A-site in NBN ceramics. Furthermore, although substantial progress has been made in enhancing the performance of NBN-based ceramics, a complete understanding of their highly complex underlying conduction mechanisms is still missing. The identification and a deeper comprehension of these mechanisms are crucial for further improving the performance of high-temperature piezoelectric materials [20,21,22]. Therefore, an accurate verification of the conduction mechanism is required for achieving excellent piezoelectric and ferroelectric characteristics in NBN-based ceramics.

In this work, the effect of LiSm co-doping at the A-site in the crystal structure, the electrical properties, and the underlying conduction mechanism of NBN-based ceramics were explored systematically. The results show that the addition of LiSm greatly improves the piezoelectric properties while maintaining a relatively high *T*_c_, and after thermal deposition at 760 °C for 1 h, the *d*_33_ still maintains 95% of its initial value, showing good thermal stability. NBN-*x*LS ceramics were mainly *p*-type materials limited by bulk conduction, and the conducting species were oxygen vacancies and holes, as revealed by impedance spectroscopy and the current–voltage characteristics.

## 2. Materials and Methods

Piezoelectric ceramics made of (NaBi)_0.5−*x*_(LiSm)*_x_*Bi_2_Nb_2_O_9_ (NBN-*x*LS, *x* = 0.00–0.06) were synthesized utilizing the conventional solid-phase technique. Stoichiometric quantities of high-purity Bi_2_O_3_ (99.99%), Sm_2_O_3_ (99.9%), Li_2_CO_3_ (99.9%), Nb_2_O_5_ (99.99%), and Na_2_CO_3_ (99.9%) were thoroughly mixed, followed by calcination for 4 h at 800 °C. The obtained powder was then finely ground, followed by its granulation with 5 wt% polyvinyl alcohol. Subsequently, the granulated powder was compressed into pellets with a diameter equal to 6 mm and a thickness equaling 1 mm at a pressure of 350 MPa. The prepared pellets were then sintered for 4 h at 1040 °C.

The crystalline structure of the NBN-*x*LS ceramics was studied via X-ray diffraction (XRD, PANalytical X-Pert PRO) characterization. The microstructure of the ceramics was studied using a scanning electron microscope (SEM, Model S-4800, Hitachi, Japan). Furthermore, the elemental distribution of the ceramics was determined through energy-dispersive spectroscopy (EDS, IE 350; INCA, Oxford, UK). High-temperature Ag pastes were coated onto two major surfaces and then heated for 30 min at 850 °C. Furthermore, a detailed analysis of the dielectric properties as a function of temperature was conducted utilizing a precision impedance analyzer (Agilent 4294A, USA). The ferroelectric hysteresis loops of the NBN-*x*LS ceramics were obtained at 150 °C using a ferroelectric test system (Precision LC, Radiant Technologies). The value of *d*_33_ was determined via a quasi-static *d*_33_ meter (Model ZJ-3A, Institute of Acoustics, Chinese Academy of Sciences, Shanghai, China). To investigate the thermal stability of the piezoelectric properties of the samples, the polarized ceramics were held at different temperatures for 1 h, and then the *d*_33_ of the annealed ceramics was measured at room temperature.

## 3. Results and Discussion

**Figure 1:**
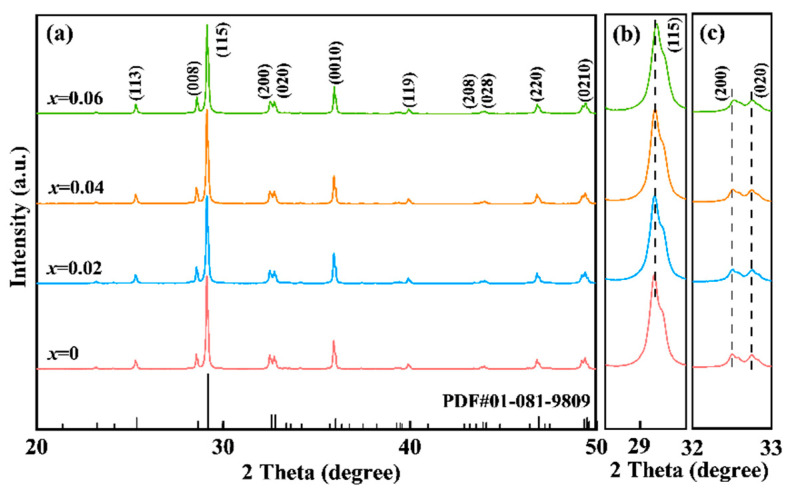
XRD patterns of NBN-*x*LS ceramics.

The XRD profiles obtained for the LiSm-doped NBN-*x*LS ceramics are presented in Figure 1. As shown in Figure 1a, the XRD peaks of all the samples were consistent with those of the standard NBN structure (PDF#01-081-9809) within a 2*θ* diffraction angle range extending from 20 to 50°. The most intense XRD signal of the prepared samples was found for the planar direction (115), without any notable impurity peaks being generated upon the incorporation of LiSm. This indicated that the NBN-*x*LS ceramic samples were single-phase ceramics with a two-layer Aurivillius structure [23,24]. The local magnification diagrams of the (200/020) and (115) crystalline planes are presented in Figure 1b,c. Note that the peaks of both crystal planes moved to higher angles with an increase in the value of *x*. The ionic radii of the Li^+^ (1.15 Å, 12 CN) and Sm^3+^ (1.24 Å, 12 CN) ions are smaller compared to those of the Na^+^ (1.39 Å, 12 CN) and Bi^3+^ (1.38 Å, 12 CN) ions [5], which caused the decrease in interplanar spacing and the constriction of cell volume.

**Figure 2:**
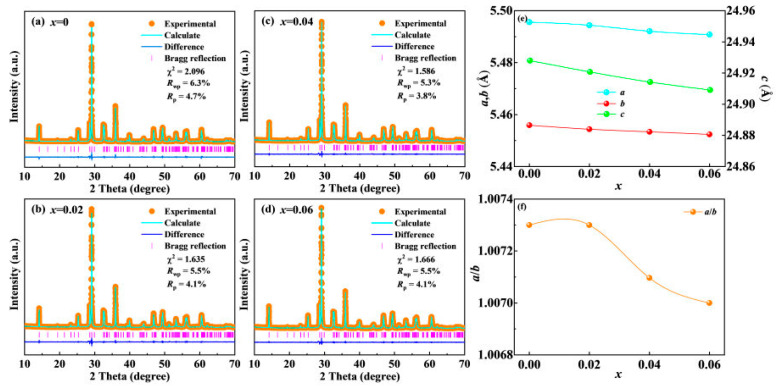
Rietveld refinements for NBN-*x*LS: (**a**) *x* = 0.00, (**b**) *x* = 0.02, (**c**) *x* = 0.04, (**d**) *x* = 0.06. (**e**) The lattice constants *a*, *b*, and *c* of NBN-*x*LS ceramics; (**f**) the *a*/*b* value.

Furthermore, XRD profiles obtained for all the components were acquired by Rietveld analysis utilizing the *A*21*am* space group in order to further evaluate the impact of LiSm doping on the crystalline structure of the (NaBi)_0.5−*x*_(LiSm)*_x_*Bi_2_Nb_2_O_9_ ceramics. The refined data obtained for each component of the NBN-*x*LS ceramics are presented in Figure 2a–d. The fitting data matched the XRD profiles quite well, and the reliability factor (*χ*^2^, *R*_wp_, and *R*_p_), quantified in the required range, showed that the refined data were reasonably accurate. The lattice constants determined from the Rietveld analysis of the XRD profiles for various components of the LiSm-doped NBN-*x*LS samples are provided in Figure 2e,f. It was found that the lattice parameters *a*, *b*, and *c* gradually decreased in value as the amount of *x* doping was raised, as shown in Figure 2e. The BLSF compounds can exist in tetragonal (*a*/*b* = 1) and orthogonal (*a*/*b* > 1) phases based on the ratio of *a*/*b* [25]. The value of *a*/*b* decreased from 1.0073 to 1.0070, further confirming that the crystalline structure of the NBN-*x*LS ceramics was transformed from an orthogonal to a pseudotetragonal phase, greatly promoting domain switching, which may enhance the piezoelectric activity [26,27].

**Figure 3:**
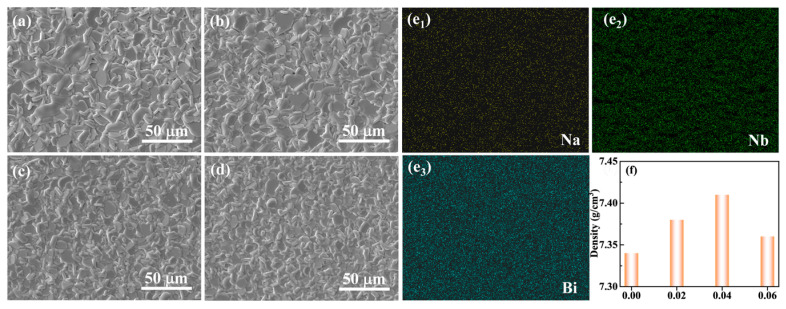
Surface microscopic morphology and elemental distribution of NBN-*x*LS ceramics with different LiSm doping amounts: (**a**) *x* = 0, (**b**) *x* = 0.02, (**c**) *x* = 0.04, (**d**) *x* = 0.06. (**e_1_**–**e_3_**) EDS mapping of elements Na, Nb, and Bi of *x* = 0 sample; (**f**) volume density of NBN-xLS ceramics.

The surface microscopic morphology and elemental composition of the NBN-*x*LS ceramics with different Sm doping amounts are presented in Figure 3. It can be determined that the crystal grains grew anisotropically due to their growth rate in the Aurivillius-structured ceramics along the *a*- and *b*-axes being higher than that along the *c*-axis. Additionally, the low surface energy of the plane in the (001) direction allowed for the formation of unique plate-like crystal grains. All the prepared samples exhibited plate-like grains, as depicted in Figure 3. The grain size of the NBN-*x*LS ceramics slowly decreased with an increase in the LiSm doping amount. The elemental distribution and composition of the *x* = 0 sample were determined via EDS mapping, the results of which are presented in Figure 3(e_1_)–(e_3_). The findings clearly showed a uniform distribution of the elements Nb, Na, and Bi. According to the Archimedes method, the densities of NBN-*x*LS ceramics are measured as displayed in Figure 3f. Note that the density of NBN-*x*LS ceramics first increases and then decreases with an increase in the LiSm doping amount.

**Figure 4:**
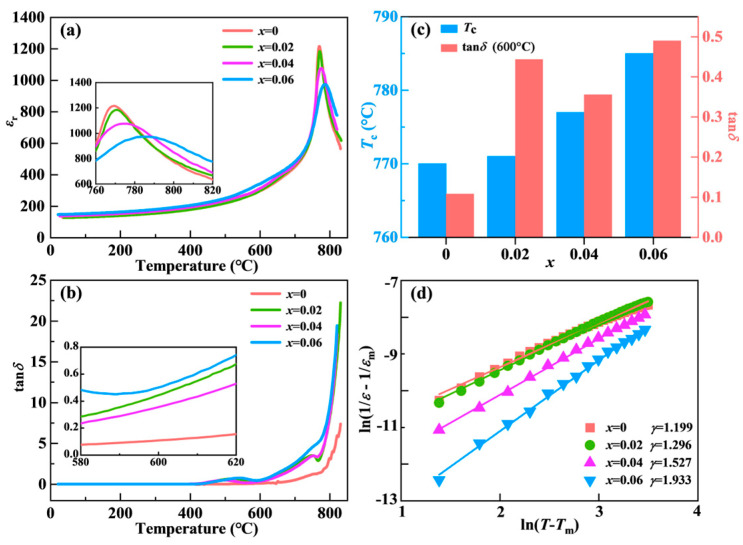
(**a**) *ε*_r_ and (**b**) tan*δ* of NBN-*x*LS ceramics, which varies in the temperature range of 25–820 °C at 500 kHz; (**c**) the *T*_c_ of each sample and the tan*δ* of each sample at 600 °C; (**d**) the relationship between log(1/*ε*–1/*ε_m_*) and log(*T*–*T_m_*) at 500 kHz.

The variation in the dielectric constant (*ε*_r_) of the NBN-*x*LS ceramics with temperature values in the range from 25 to 820 °C is displayed in Figure 4a. The existence of two dielectric anomalies, occurring at 400−600 °C and 750−800 °C, can be observed. The dielectric anomaly within the range of 400−600 °C may be associated with the defect-related dielectric response [11]. The dielectric anomaly within the range of 750−800 °C corresponds to a phase transition from the ferroelectric orthorhombic phase to the paraelectric tetragonal phase. The temperature corresponding to the maximum value of *ε*_r_ represents the value of *T*_c_. Note that the value of *T*_c_ gradually increased from 770 to 785 °C with an increase in the doping amount of LiSm, with the *T*_c_ value for the NBN-0.04LS ceramic being equal to 777 °C, as shown in Figure 4c. The substitution of the asymmetric Bi^3+^ ion with the symmetric Sm^3+^ ion should, in theory, reduce the orthorhombic distortion, thereby decreasing the value of *T*_c_. Also, the replacement of the Na^+^ ion by the smaller Li^+^ ion can lead to an enhancement in lattice distortion, increasing the *T*_c_ value. Therefore, the tolerance factor (*t*) of the perovskites was calculated to obtain a better evaluation of the impact of Li^+^ and Sm^3+^ co-substitution on the value of *T*_c_. The mathematical relationship of *t* for the perovskite-layer units (AB_2_O_7_)^2−^ can be described as follows [28]:

(1) $$t=(r_{A}+r_{O})/\surd 2(r_{B}+r_{O})$$

where *r_O_*_,_ *r_A_*, and *r_B_* denote the ionic radii of the oxygen ion, A-site cation, and B-site cations, respectively. Based on Equation (1), it is clear that the value of *t* should decrease with a reduction in the A-site cationic radius. Therefore, the increase in *T*_c_ can be associated with the decrease in *t* within the NBN-*x*LS ceramics. This feature was consistent with that exhibited by the Aurivillius oxide MeBi_2_Nb_2_O_9_ (Me = Ca, Sr, and Ba) [28]. The tan*δ* values of all the LiSm-doped NBN samples at 600 °C is much higher than that of the pure NBN ceramic. When *x* = 0.04, tanδ reaches its lowest value. This reduced tan*δ* value may be related to the decrease in oxygen vacancies [24]. Additionally, the dielectric peak widened with an increase in the LiSm doping amount. This was ascribed to the cation disorder caused by LiSm replacement within the crystalline structure of the NBN-*x*LS ceramics. This effect was also observed in the LiCe-doped NBN and NaCe-doped CaBi_2_Nb_2_O_9_ [29,30] structures. The temperature dependence of the dielectric loss (tan*δ*) for the NBN-*x*LS ceramics is illustrated in Figure 4b. The value of tan*δ* for all the obtained samples remained relatively low for temperatures below 600 °C. However, its value increased sharply as the temperature was increased beyond this point. The results presented in Figure 4c show that the value of tan*δ* for the NBN-*x*LS ceramics doped by LiSm was larger than that of the pure NBN sample at 600 °C. This may be associated with the increase in defect concentration within the prepared ceramics [24].

The dielectric behavior in normal ferroelectrics follows the Curie–Weiss law for temperatures above *T*_c_, which may be ascribed to the dielectric relaxation phenomenon. Therefore, a modified Curie–Weiss law was employed to describe it, as given below [31,32]:

(2) $$\frac{1}{\varepsilon }-\frac{1}{\varepsilon_{m}}=\frac{{\left(T-T_{m}\right)}^{\gamma }}{C}$$

where *ε_m_* denotes the maximum dielectric constant, *T_m_* represents the temperature corresponding to the dielectric peak, *C* corresponds to the Curie constant, and *γ* indicates the dispersion factor, with its value ranging from 1 for normal ferroelectric to 2 for ideal ferroelectric relaxation. As depicted in Figure 4d, the value of *γ* for the NBN-*x*LS ceramics gradually increased from 1.199 to 1.933 with an increase in the LiSm doping amount, displaying the features of a dispersive phase transition. Also, the dispersion degree slowly increased, suggesting that all samples exhibited dielectric relaxation properties [33].

**Figure 5:**
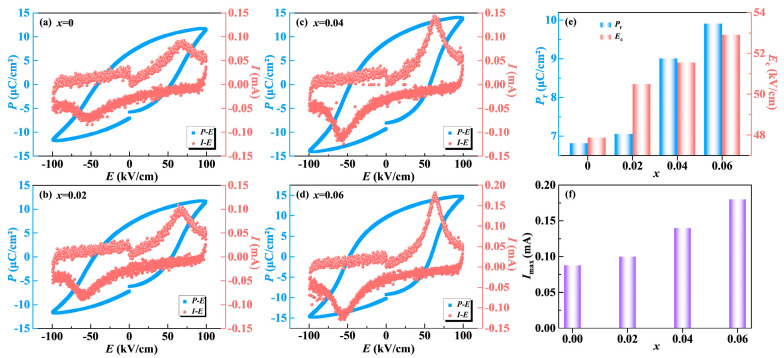
(**a**–**d**) *P-E* and *I-E* loops of NBN-*x*LS ceramics measured at 150 °C and 10 Hz; (**e**) the composition dependence of *P*_r_ and *E*_c_; (**f**) the current peak (*I*_max_) as a function of *x*.

The ferroelectric polarization-electric field (*P*-*E*) and current-electric field (*I*-*E*) loops obtained for the NBN-*x*LS ceramics at 150 °C and 10 Hz are presented in Figure 5a–d. The *P*-*E* loop of the pure NBN ceramic exhibited a flat and narrow shape, suggesting poor ferroelectric characteristics. Conversely, the *P*-*E* loops of the LiSm-doped specimens were more saturated, indicating that LiSm doping substantially improved the ferroelectric properties. As depicted in Figure 5e, the remanent polarization (*P*_r_) monotonously enhanced with an increase in the value of *x*, reaching a maximum value equal to 10.6 μC/cm^2^ when *x* = 0.06, which was much higher compared to the value obtained for the pure NBN ceramic at 6.85 μC/cm^2^. The increased *P*_r_ value can be attributed to reduced orthogonal distortion, which facilitates enhanced separation of positive and negative charge centers in the NbO_6_ octahedron, thereby improving polarizability. This result coincides with the decrease in the *a*/*b* value obtained from XRD Rietveld refinements in Figure 2f.

In general, spontaneous polarization reversal occurs near the coercive field (*E*_c_), resulting in the appearance of a switching current peak (*I*_max_) in the *I-E* curves [34]. The value of *I*_max_ is closely linked to the polarization switching properties and can be utilized to assess the domain switching/reorientation behavior in the applied field [35]. It was observed that LiSm-doped samples exhibited intense current peaks in the *I-E* curves, suggesting a lower energy barrier for ferroelectric domain switching. As illustrated in Figure 5f, the *x* = 0.06 sample displayed the highest *I*_max_ value, which suggested that it demonstrated the most effective domain switching.

**Figure 6:**
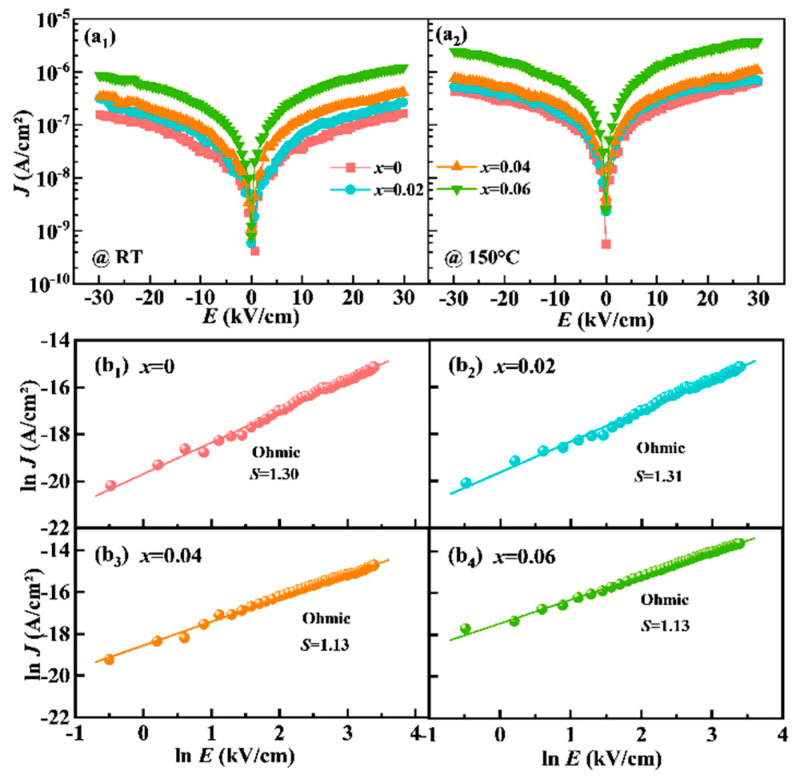
Leakage current density and conduction mechanism of NBN-*x*LS ceramics. *J-E* curves measured at (**a_1_**) RT and (**a_2_**) 150 °C; (**b_1_**–**b_4_**) ln*J*-ln*E* curves at RT.

A deep understanding of the conduction mechanism is essential for improving the electrical efficiency further. The graphs of the leakage current density (*J*) plotted against the applied field (*E*) for the NBN-*x*LS ceramics measured in the same electric field at room temperature (RT) are presented in Figure 6(a_1_), where the sample thickness is slightly different. Note that the value of *J* for the *x* = 0.06 sample was enhanced by more than one order of magnitude compared to that of the pure NBN ceramic at RT. This enhanced *J* value may be related to the increase in the concentration of oxygen vacancies. Furthermore, the value of *J* increased slightly at 150 °C compared to that at RT, as shown in Figure 6(a_2_). No significant difference in the *J* value was observed when the bias was reversed, indicating that bulk-limited conduction was the fundamental mechanism at play within the NBN-*x*LS ceramics. The logarithmic plots of *J* as a function of *E* are presented in Figure 6(b_1_)–(b_4_). The log(J) vs. log(E) curves obtained for the NBN-*x*LS ceramics were linear, closely following the ohmic law, with their slopes exhibiting a value of nearly 1. Therefore, ohmic conduction was the dominant conduction mechanism in the NBN-*x*LS ceramic samples. This suggested that the thermally produced free carrier density inside the samples was larger than the injected carrier density [36]. Such behavior occurs in the quasi-electric neutral state in which partial trap centers are filled under weak injection.

**Figure 7:**
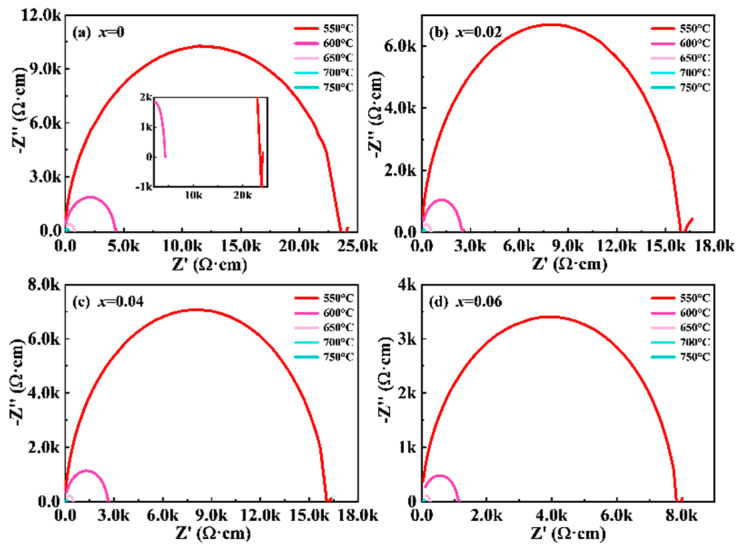
The Cole-Cole curves of NBN-*x*LS ceramics over 550–750 °C.

Furthermore, the NBN-*x*LS samples were analyzed via complex impedance spectroscopy to obtain a deeper understanding of the conduction mechanisms, as shown in Figure 7. The Cole–Cole curves of the NBN-*x*LS ceramics were determined over a temperature range extending from 550 to 750 °C. The radius of the impedance semicircle decreased with an increase in the value of *x*, implying an enhancement in electrical conductivity. The kinetic energy of the electrons increases upon thermal excitation at high temperatures, leading to their ejection from the nucleus as free electrons. This phenomenon increased the electrical conductivity of the LiSm-doped ceramics through the generation of a high concentration of oxygen vacancies and holes. Electrical conduction is primarily dominated by the grains at high frequencies. Furthermore, as the temperature rises, the conductive mechanism changes. At 550 °C, impedance spectra exhibited an additional low-frequency feature, which displayed the characteristics of an inclined Warburg spike, suggesting the appearance of weak ionic conduction [37,38], as shown in the inset of Figure 7a. Therefore, the grain component contributed to the conductivity of all the ceramics and the occurrence of weak ion conduction at high temperatures.

**Figure 8:**
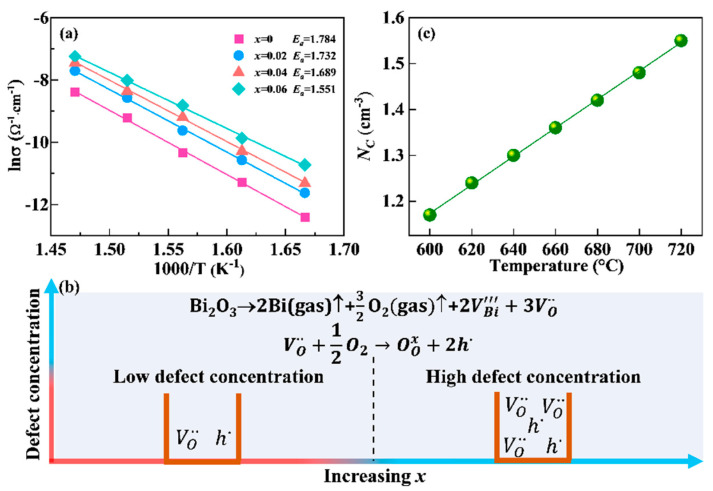
(**a**) Arrhenius plots for NBN-*x*LS ceramics over 600–680 °C; (**b**) temperature dependence of the effective density of states of the conduction band (*N*_C_) in a low-resistance state in NBN-*x*LS ceramics; (**c**) schematic of the formation of oxygen vacancies ($V_{O}^{\cdot \cdot }$ and holes ($h^{\cdot }$), where increasing *x* makes the defect concentration increase.

In general, the carrier concentration is closely linked to the resistance value of the ceramic material. The drop in the value of resistance for the LiSm-doped NBN-*x*LS samples may be ascribed to the rise in the concentration of holes and oxygen vacancies. The activation energy *E_a_* can be determined using the Arrhenius equation given below:

(3) $$\sigma =\sigma_{0}exp(-E_{a}/kT)$$

where *E_a_* denotes the conductivity activation energy, *T* represents the absolute temperature, *σ_0_* indicates the pre-exponential factor, and *k* denotes Boltzmann’s constant. The value of *E_a_* was determined by linear fitting of the obtained data via Equation (3), as depicted in Figure 8a. The *E_a_* value for the pure NBN ceramic was found to be 1.784 eV, corresponding to its intrinsic conductance. The *E_a_* value obtained for the LiSm-doped ceramics was between 1.551 and 1.732 eV. An apparent reduction in the value of *E_a_* was seen with an increase in the LiSm doping amount, which was linked to the rise in the concentration of defects, such as oxygen vacancies and holes. This can explain the increase in the number of carriers and the reduction in the insulation of the samples. Therefore, results proved that a *p*-type conduction mechanism was the dominant mechanism within the NBN-*x*LS ceramics. This was consistent with the reduction in impedance (Figure 7) and the increase in dielectric loss (Figure 4c). The generation of oxygen vacancies ($V_{O}^{\cdot \cdot }$) and holes ($h^{\cdot }$) can be described by the defect equations in Figure 8b.

(4) $$Bi_{2}O_{3}\to 2Bi(gas)↑+\frac{3}{2}O_{2}(gas)↑+2V_{Bi}^{\prime \prime \prime }+3V_{O}^{\cdot \cdot }$$

(5) $$V_{O}^{\cdot \cdot }+\frac{1}{2}O_{2}\to O_{O}^{x}+2h^{\cdot }$$

Several important physical parameters can be determined in the NBN-*x*LS ceramics based on the analyses of the bulk-limited conduction mechanisms, including the effective density of the states of the conduction band (*N*_C_) and the carrier drift mobility. The parameter *N*_C_ is dependent on temperature, according to the relationship *βT*^3/2^, where *β* is a constant [39]. The value of *N*_C_ in the NBN-*x*LS ceramics can be obtained, as shown in Figure 8c. The *N*_C_ value in the NBN-*x*LS ceramics at 600 °C was found to be 1.17 × 10^19^ cm^−3^.

**Figure 9:**
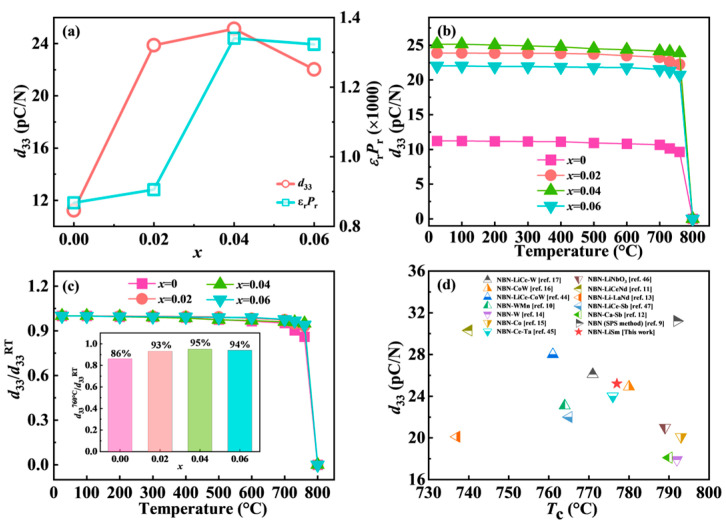
(**a**) Composition dependence of *d*_33_ and *ε*_r_*P*_r_; (**b**) thermal stability of *d*_33_; (**c**) relationship between normalized *d*_33_ and annealing temperature, with the inset representing the composition dependence of normalized *d*_33_ at 760 °C; (**d**) a comparison of *T*_c_ and *d*_33_ values reported in this work and for other NBN-based ceramics.

The composition dependence of the *d*_33_ value for the NBN-*x*LS ceramics is illustrated in Figure 9a. It was evident that moderate LiSm doping improved the piezoelectric characteristics of the NBN ceramics. The value of *d*_33_ for the piezoelectric materials was estimated from the product of *ε*_r_ and *P*_r_ based on their empirical equation (*d*_33_ = 2*Qε*_0_*ε*_r_*P*_r_) [40,41]. The values of *d*_33_ for the NBN-*x*LS ceramics were measured using a quasi-static *d*_33_ meter (Model ZJ-3A) at RT. The variation in the estimated *d*_33_ value is the same as that measured in the NBN-*x*LS ceramics. The *d*_33_ value of the NBN-*x*LS ceramics rose initially and then decreased with the increase in the LiSm doping amount, reaching a maximum value equal to 25.2 pC/N at *x* = 0.04, which coincides with the change in the density displayed in Figure 3f. The improvement in the *d*_33_ value can be attributed to the reduced distortion, which lowers the energy barrier for NbO_6_ octahedron tilting and facilitates polarization rotation under electric fields.

The temperature stability of the piezoelectric materials affected their potential applications. To evaluate the thermal stability of the piezoelectric properties of the NBN-*x*LS samples, the polarized ceramics were held at different temperatures for 1 h, and then the *d*_33_ of the annealed ceramics was measured at RT, as shown in Figure 9b,c. The values of *d*_33_ obtained for the samples exhibited a slight reduction for temperatures below 600 °C. This was associated with the presence of unstable non-180° domain walls within the synthesized ceramics [42]. These domains return to their original unstable states due to the combined influences of internal stress and the thermal field, resulting in a slight deterioration in the piezoelectric characteristics [43]. The *d*_33_ value for all samples fell sharply for temperatures exceeding 700 °C. For temperature values beyond the *T*_c_ value, the value of *d*_33_ decreased rapidly to 0. Piezoelectric materials generally undergo a ferroelectric-to-paraelectric phase transition near the *T*_c_ value, leading to a loss in their piezoelectric response. The compositional dependence of the ratio value of *d*_33_ at 760 °C (*d*_33_^760 °C^) to that at RT (*d*_33_^RT^) is presented in the inset of Figure 9c. The NBN-0.04LS ceramic maintains 95% of its original *d*_33_ value after 760 °C annealing, which is critical for sensor durability under harsh conditions. This indicates that LiSm doping substantially improved the temperature stability of the NBN samples. Figure 9d shows a comparison of *T*_c_ and *d*_33_ values reported in this work and in other NBN-based ceramic correlation reports [44,45,46,47]. It can be seen that LiSm co-doped NBN ceramics simultaneously have higher *d*_33_ and *T*_c_ values, indicating that the ceramics have potential for use in high-temperature devices.

## 4. Conclusions

The impact of LiSm co-doping on the structure, electrical characteristics, and conduction mechanisms of NBN piezoelectric ceramics was studied systematically. The lattice distortion reduced with an increase in the LiSm doping amount, resulting in improved ferroelectric and piezoelectric properties. The NBN-0.04LS ceramics displayed the best performance, achieving *T*_c_, *P*_r_, and *d*_33_ values of 777 °C, 9.05 μC/cm^2^, and 25.2 pC/N, respectively. Furthermore, NBN-0.04LS ceramics retained 95% of their original *d*_33_ value after high-temperature annealing. *p*-type conduction is dominant and the conducting species were oxygen vacancies and holes. The grain component contributed to the conductivity and the occurrence of weak ion conduction at high temperatures. These results demonstrate that LiSm co-doped NBN ceramics have potential for use in high-temperature sensors.

## Data Availability

The original contributions presented in this study are included in the article. Further inquiries can be directed to the corresponding authors.
